# Supplementary material for: Oligomeric Proanthocyanidins Ameliorate Cadmium-Induced Senescence of Osteocytes Through Combating Oxidative Stress and Inflammation
Source: Antioxidants (Basel). 2024 Dec 12;13(12):1515. doi: 10.3390/antiox13121515 (PMC11727362; doi:10.3390/antiox13121515)
Supplement: Supplementary file 1 [file antioxidants-13-01515-s001.zip › antioxidants-3278416-supplementary.pdf]

Supplementary Table S1. The primer sequences were used for qRT-PCR

Gene name	Primer sequences (5–3')	Gene number	Length (bp)
Nrf2	Forward: GCCACCGCCAGGACTACAG<br>Reverse: AACTTGTACCGCCTCGTCTGG	NM_001399226.1	84
NQO1	Forward: CTGAAGAAGAGAGGATGGGAGGTAC<br>Reverse: TGCTAGAGATGACTCGGAAGGATAC	XM_036153810.1	138
GCLC	Forward: GCACATCTACCACGCAGTCAAG<br>Reverse: ACATCGCCTCCATTCAGTAACAAC	NM_010295.2	129
GCLM	Forward: TGACTCACAATGACCCGAAAGAAC<br>Reverse: AGTACCTCAGCAGCCACAGC	NM_008129.4	117
β-actin	Forward: TATGCTCTCCCTCACGCCATCC<br>Reverse: GTCACGCACGATTTCCCTCTCAG	NM_007393.5	129

Figure S1

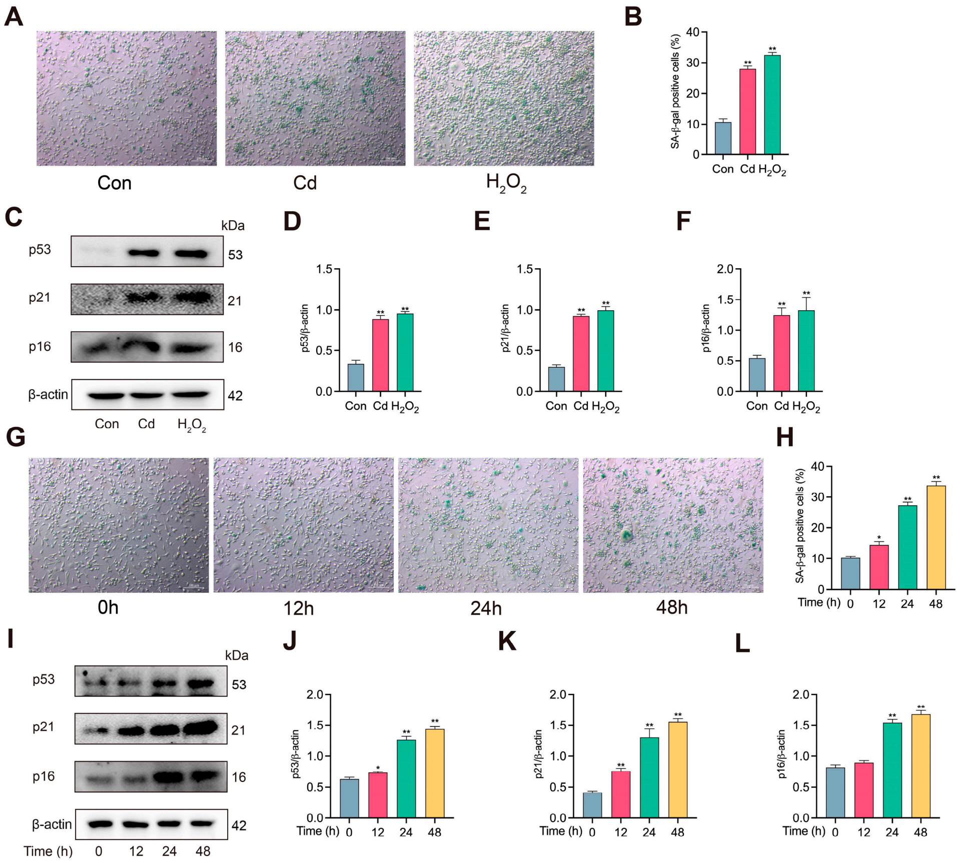

Figure S1. Cd exposure induces senescence of osteocytes. MLO-Y4 cells were treated with 6 μmol/L Cd for 24 h, or treated with 200 μmol/L H_2O_2 for 2 h and then cultured for 24 h. Cellular senescence was detected by SA-β-gal staining (A and B). Scale bar = 100 μm. The protein expression of p53, p21 and p16 was detected by western blot (C-F). MLO-Y4 cells were treated with 6 μmol/L Cd for different times. Cellular senescence was detected by SA-β-gal staining (G and H). Scale bar = 100 μm. The protein expression of p53, p21 and p16 was detected by western blot (I-L). Results are shown as the mean ± SD (n = 3). Compared with the control group, $^{*}P < 0.05$, $^{**}P < 0.01$.

Figure S2

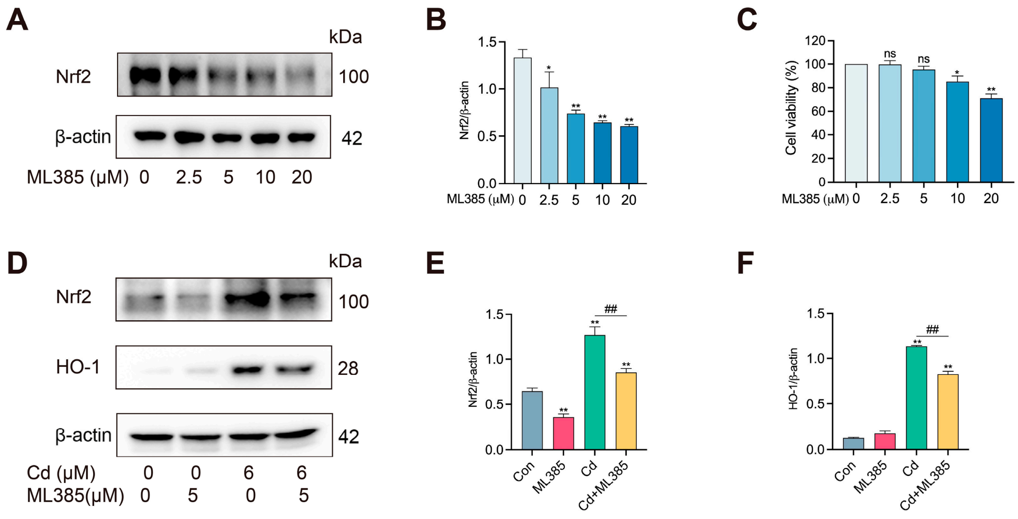

Figure S2. Effect of the Nrf2 inhibitor ML385 on Cd-induced elevation of Nrf2 activity in osteocytes. MLO-Y4 cells were treated with different concentrations of ML385 for 24 h. The protein expression of Nrf2 was detected by western blot (A and B). Cell viability was evaluated using the CCK-8 assay (C). MLO-Y4 cells were co-treated with 6 μmol/L Cd and 5 μmol/L ML385 for 24 h. The protein expression of Nrf2 and HO-1 was detected by western blot (D-F). Results are shown as the mean ± SD (n = 3). Compared with the control group, * $P < 0.05$, ** $P < 0.0$. Compared with the Cd group, $^{\#\#}$ $P < 0.01$.
